# Valorization of Tomato Agricultural Waste for 3D-Printed Polymer Composites Based on Poly(lactic acid)

**DOI:** 10.3390/polym16111536

**Published:** 2024-05-29

**Authors:** Sotirios Pemas, Dimitrios Gkiliopoulos, Christina Samiotaki, Dimitrios N. Bikiaris, Zoi Terzopoulou, Eleftheria Maria Pechlivani

**Affiliations:** 1 Centre for Research and Technology Hellas, Information Technologies Institute, 6th km Charilaou-Thermi Road, 57001 Thessaloniki, Greece; sopemas@iti.gr (S.P.); dgiliopo@chem.auth.gr (D.G.); 2 Laboratory of Chemical and Environmental Technology, Department of Chemistry, Aristotle University of Thessaloniki, 54124 Thessaloniki, Greece; 3 Laboratory of Chemistry and Technology of Polymers and Colors, Department of Chemistry, Aristotle University of Thessaloniki, 54124 Thessaloniki, Greece; samiotaki@chem.auth.gr (C.S.); dbic@chem.auth.gr (D.N.B.); 4 Laboratory of Industrial Chemistry, Department of Chemistry, University of Ioannina, 45110 Ioannina, Greece

**Keywords:** additive manufacturing (AM), 3D printing, fused filament fabrication (FFF), filament, poly(lactic acid) (PLA), mechanical properties, tomato stem, circular economy (CE), agricultural waste, agricultural waste management

## Abstract

Agricultural waste is a renewable source of lignocellulosic components, which can be processed in a variety of ways to yield added-value materials for various applications, e.g., polymer composites. However, most lignocellulosic biomass is incinerated for energy. Typically, agricultural waste is left to decompose in the fields, causing problems such as greenhouse gas release, attracting insects and rodents, and impacting soil fertility. This study aims to valorise nonedible tomato waste with no commercial value in Additive Manufacturing (AM) to create sustainable, cost-effective and added-value PLA composites. Fused Filament Fabrication (FFF) filaments with 5 and 10 wt.% tomato stem powder (TSP) were developed, and 3D-printed specimens were tested. Mechanical testing showed consistent tensile properties with 5% TSP addition, while flexural strength decreased, possibly due to void formation. Dynamic mechanical analysis (DMA) indicated changes in storage modulus and damping factor with TSP addition. Notably, the composites exhibited antioxidant activity, increasing with higher TSP content. These findings underscore the potential of agricultural waste utilization in FFF, offering insights into greener waste management practices and addressing challenges in mechanical performance and material compatibility. This research highlights the viability of integrating agricultural waste into filament-based AM, contributing to sustainable agricultural practices and promoting circular economy initiatives.

## 1. Introduction

The circular economy (CE) is gaining significant momentum as a solution to diverse socioenvironmental challenges [1,2]. The CE aims to reduce material consumption, minimize waste, and shift away from the linear economy model, which is unsustainable and reliant on fossil fuels [3]. To address this, the CE promotes closed-loop systems for material and energy flow to eliminate waste [3,4]. This transition is facilitated through strategies known as the R-imperatives. Initially, the 3Rs (Reduce, Reuse, Recycle) were prominent, but the growing concept of 10R-imperatives incorporates additional principles like refurbish and repair [3,5,6]. These imperatives can be applied across various industries, including manufacturing, construction, maritime, food, and agriculture, where current processes often lack protocols for waste utilization [7].

Especially for agricultural waste valorization, it is crucial to develop new sustainable methods for processing the tons of waste produced annually [8,9]. Due to factors like population growth, agricultural production has increased, resulting in approximately 998 million tons of agricultural waste generated each year [8,10]. This substantial amount cannot be naturally decomposed and has negative impacts on soil, air, and water resources. Additionally, conventional agricultural waste treatment methods, such as burning, can worsen air pollution [10,11,12,13,14].

In recent decades, there has been a growing awareness of the need for more efficient circular economy models to mitigate the environmental impact of human activities, particularly in agricultural waste generation [9,15]. However, amidst these challenges lies an opportunity for innovation, especially within the domain of additive manufacturing (AM). By harnessing agricultural waste as a resource for 3D printing, not only are the harmful effects of agricultural waste accumulations mitigated, but new avenues for exploring composite materials are also unlocked [16,17,18].

AM technologies offer extensive possibilities for utilizing a wide range of feedstock materials, whether virgin or recycled, while at the same time maintaining their cost effectiveness in technologies such as Fused Filament Fabrication (FFF) [16,19,20]. This flexibility allows for the creation of various material combinations, constantly expanding the range of available composite materials [18,21]. With this potential, AM offers a greener approach to waste exploitation. Particularly, as mentioned earlier, agricultural waste contributes significantly to planetary pollution and can, alternatively, be utilized to create value-added secondary life products [3,9,22]. Agricultural waste can be effectively incorporated into composite materials, enhancing their properties and promoting better waste management practices. Furthermore, especially for biodegradable materials, agricultural waste is a valuable asset for enhancing properties while maintaining biodegradability [17,23,24]. The conversion of agricultural waste into feedstock material for AM technologies can be achieved through numerous systems that have emerged in the last decade, providing the capability to convert used, recycled, and scrap materials into feedstock for 3D printing, typically in pellet or filament form [25,26].

For utilizing waste in AM, the most common methods found in the literature involve converting waste into pellets or granules. These pellets are then used in filament development systems to create filament, which is subsequently used in FFF 3D printers [26,27,28]. Alternatively, pellets can be directly used in 3D printers that use them as feedstock material. This direct method, known as Fused Particle Fabrication (FPF) or Fused Granular Fabrication (FGF), involves pushing pellets through a heated nozzle using a screw, where they are extruded as melted material [29,30].

Andrzejewski et al. [23] developed a composite material suitable for Fused Deposition Modelling (FDM) and employed buckwheat husk (BH) particles as a demonstration of utilizing lignocellulosic waste. This study validated the printability of a composite material containing agricultural waste particles and investigated the mechanical properties of PLA-based composites incorporating BH particles, poly(butylene adipate-co-terephthalate) (PBAT), and thermoplastic starch (TPS). Bahçegül et al. [24] investigated a novel approach enabling 3D printing of hemicellulosic polymers extracted from lignocellulosic agricultural wastes (corn cobs) without any chemical modifications or blending with another polymer. Moreover, by printing a scaffold prototype, they demonstrated the potential of the underutilized hemicellulose biopolymer in biomedical applications. Acevedo et al. [31] explored the significance of combining CE with agricultural practices by repurposing agricultural waste. They present the manufacturing of FDM filaments from various materials like kenaf fibres, cork residues, rice, vine shoots, paulownia, poplar, and thistle, along with bars of PLA mixed with kenaf fibre through an extrusion process. Scaffaro et al. [18] presented the 3D printability of green composites using lignocellulosic fillers from Opuntia ficus indica and Posidonia oceanica in PLA via FDM. It was found that up to 20% of bioplastic can be replaced with these ecofriendly fillers without significant variations in mechanical performance. Additionally, an increase in surface hydrophilicity was observed, potentially enhancing biodegradability postdisposal.

Although many attempts are found in the literature for utilizing agricultural waste for various applications [32,33,34,35], not much research exists on using it to create composites via 3D printing. This study enriches the possibilities of valorising agricultural waste in the development of new materials that can be used by 3D printing technologies, aiming to replace petroleum-based nonrecycled and nonenvironmentally friendly materials. More specifically, this study illustrates an innovative approach to integrate agricultural waste management via CE standards through FFF additive manufacturing technology, thus decreasing the cost, weight and environmental impact of fully plastic filaments. Tomato stems were selected as the raw material, due to their local availability as well as their potential for antimicrobial and antioxidant activities due to their phenolic acids and flavonoids content [36]. Tomatoes are widely cultivated globally, particularly in the Mediterranean area of the European Union (EU), where hectares of land are dedicated to their growth across countries like Spain, Italy, Portugal, and Greece. According to the Food and Agriculture Organization of the United Nations (FAO), in 2022, tomatoes emerged as the most abundantly produced vegetable, reaching an impressive 186 million tonnes [37]. Tomato stems are a byproduct of tomato cultivation and are rich in biopolymers such as cellulose, hemicellulose, and lignin [38,39]. They are often discarded and burned or pyrolyzed to produce energy, therefore, utilizing this byproduct in other ways would be of great interest to various industry sectors in many countries worldwide [39,40,41,42]. An alternative application for tomato stems could be the AM. 3D-printed polymer/biomass composites have shown similar properties to neat polymers [43,44], while their woody texture makes them ideal for specific applications, such as furniture and interior design. Especially, in AM, wood-based 3D printing filaments, as well as wood-derived 3D printable materials (cellulose-based, hemicellulose-based, lignin-based materials), can be utilized in various state-of-the-art applications, such as encapsulation in devices for improved electromagnetic interference shielding [45,46].

To demonstrate the printability of tomato agricultural waste in AM applications, tomato stems were processed, and converted into powder form, along with Joncryl as a chain extender and poly(lactic acid) (PLA), a widely recognized biobased and compostable polymer in the 3D printing industry [47,48]. Two different mixtures were created, resulting in two FFF filaments: 1. PLA/Joncryl/5% tomato stem powder filament and 2. PLA/Joncryl/10% tomato stem powder filament. Additionally, a reference point is established through the development of a third filament consisting solely of PLA and Joncryl: 3. PLA/Joncryl filament. These filaments were used as 3D printing feedstock materials to produce specimens for testing their printability, physicochemical, mechanical properties as well as their antioxidant activity.

**Figure 1:**
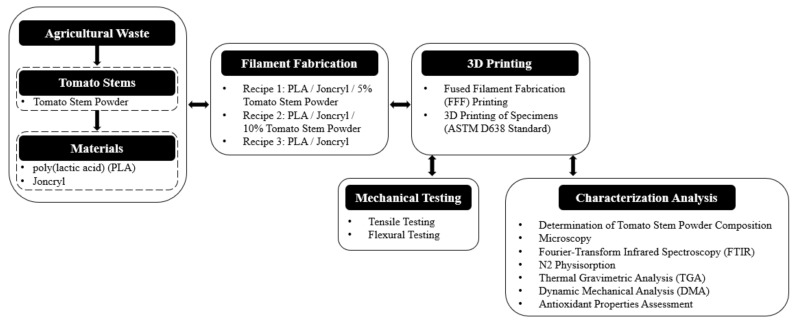
Architectural diagram illustrating the structure of the present study.

This paper is organized as follows: Section 2 outlines the materials and methods employed in the development and assessment of the present study. Section 3 presents the results and exhibits the properties of 3D-printed composite specimens incorporating tomato stem powder as agricultural waste. Finally, Section 4 includes the conclusions of the study. Figure 1 presents the architectural diagram of the present study’s methodology to obtain assessments and conclude if the proposed agricultural waste exploitation scenario for 3D printing applications is feasible.

## 2. Materials and Methods

### 2.1. Materials

**Figure 2:**
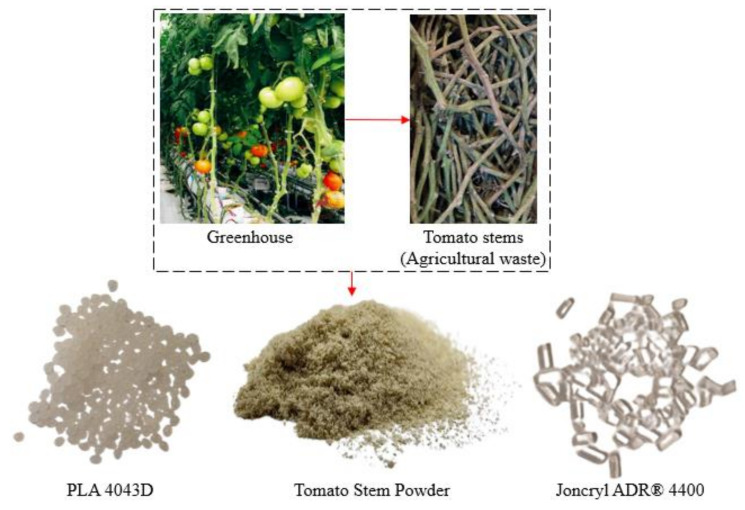
Experimental Materials.

In this study, the filament’s development involved a combination of PLA in pellet form, Joncryl as a chain extender, and tomato stem powder with particles smaller than 150 μm. The PLA pellets used were of the PLA 4043D type, supplied by 3devo (Utrecht, The Netherlands). The chain extender, Joncryl ADR^®^ 4400, was provided by BASF (Ludwigshafen, Germany), with an epoxy equivalent weight of 485 g/mol and a weight-average molecular weight of 7100 g/mol. The tomato stems (*Solanum lycopersicum* L.) were cultivated in 2023, in the greenhouse developed within the EU Green Deal project PestNu, funded by the European Union’s Horizon 2020 research and innovation programme. Figure 2 illustrates the experimental materials used in the present study.

### 2.2. Production of Tomato Stem Powder (TSP)

Tomato plant pruning was turned into fine powder via a knife mill (Moulinex LM811 household blender, 1200 W, Groupe SEB, Ecully, France). First, tomato stems were chopped into small pieces, and then dried in a vacuum oven at 80 °C overnight. The dried pieces were then milled using a knife mill at half speed (approximately 14,000 rpm) for 30 min, resulting in fibrous particles. The fibrous particles were redried under vacuum at 80 °C until complete removal of moisture, followed by a second milling at the same conditions, resulting in a fine-pow-dered product. The powder fraction with particle size smaller than 150 μm was separated using a proper sieve and was further used to produce polymer composite filaments.

### 2.3. Development Process of the PLA Tomato Stem Filaments

For the development of the filaments, PLA pellets were first vacuum-dried overnight at 40 °C [27]. Subsequently, the dried PLA pellets were mixed with Joncryl, contributing to improved physicochemical properties in the resulting filament [27,49] and tomato stem powder in different proportions to create two distinctive filaments. Both filaments consisted of 2% Joncryl, with one including 5% tomato stem powder (referred to as PLA-5%TSP) and the other 10% tomato stem powder (referred to as PLA-10%TSP).

Specifically, the composition of the filament named PLA-5%TSP includes 93% (wt.%) PLA, 2% (wt.%) Joncryl, and 5% (wt.%) tomato stem powder. Meanwhile, the filament named PLA-10%TSP includes 88% (wt.%) PLA, 2% (wt.%) Joncryl, and 10% (wt.%) tomato stem powder. Finally, the composition of the third filament, referred to as PLA, includes 98 wt.% PLA and 2 wt.% Joncryl.

The 3D printing filaments were developed using the 3devo Composer Series 350/450 filament maker (Utrecht, The Netherlands). The fluctuations in the final filaments’ diameters were within ±0.07. The primary objective was to achieve a 1.75 mm diameter filament. However, due to the heterogeneity of the mixtures, the diameters ranged between 1.68 mm and 1.82 mm. Only filaments with a diameter within this range were used, as it was observed that this variation did not pose problems in the subsequent 3D printing process.

**Figure 3:**
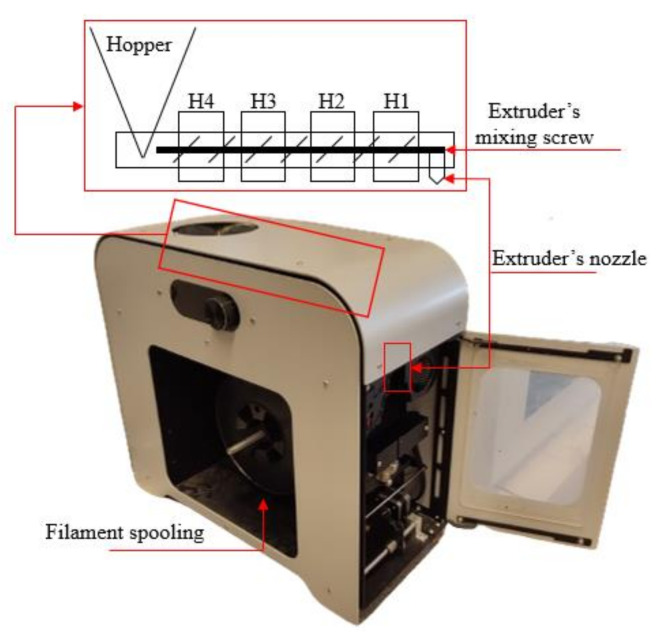
Illustration of the 3devo Filament Maker Parts.

The filament maker machine, illustrated in detail in Figure 3, was configured with four different temperatures across its four distinct heating zones. The purpose of these heating zones is to melt and homogenize the feedstock material (in this case, PLA pellets, Joncryl, and tomato stem powder) and, with an integrated mixing screw, propel the melted material through a nozzle for filament development. To achieve the optimal temperature combination, it was set as follows: Heater 1 (H1)—165 °C, Heater 2 (H2)—175 °C, Heater 3 (H3)—175 °C, and Heater 4 (H4)—170 °C. Initially, the feedstock material passes through Heater 4, which is closest to the feedstock hopper, where the materials in pellet form are inserted. It then progresses through the other heating zones (Heater 3, Heater 2, and Heater 1) before reaching the nozzle. Regarding the extruder’s screw rotational speed, driving material through the heating zones and nozzle, it was set at 4.5 rpm, and the integrated fans were adjusted to 90% for the solidification of the filament during the fabrication process.

### 2.4. Additive Manufacturing Process

**Figure 4:**
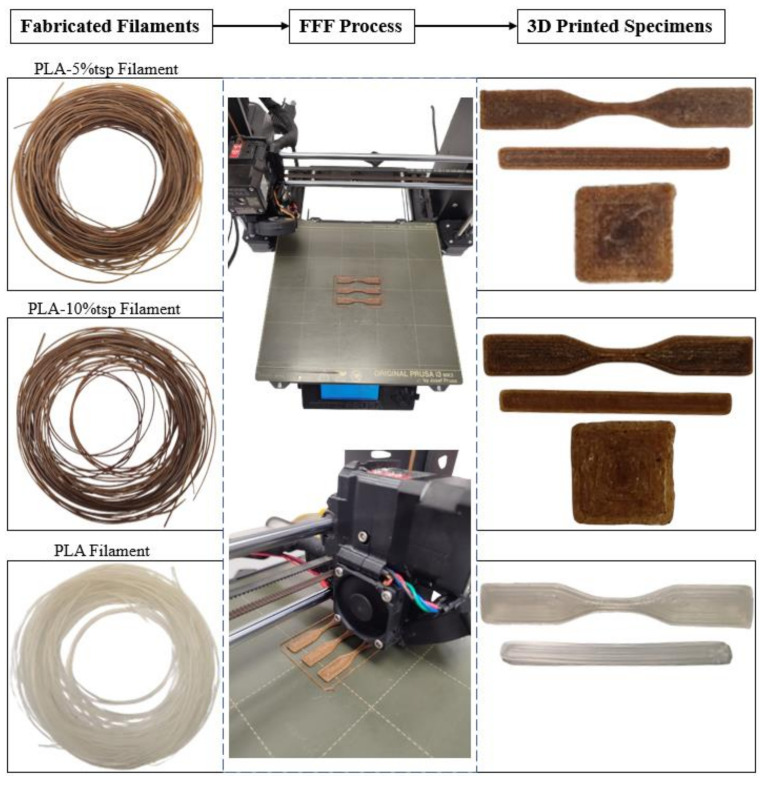
Visual Overview of the Additive Manufacturing Process.

For the investigation of the developed filament’s printability, FFF technology was utilized. In addition to printability assessments, specific specimens were designed using SOLIDWORKS^®^ CAD Software (2022 SP2.0 Professional version) to conduct mechanical property tests and material characterization. These designed specimens were 3D-printed using an Original Prusa i3 MK3S+ 3D printer and Prusa Slicer 2.5.2 software. From the three different developed filaments (PLA-5%TSP, PLA-10%TSP and PLA), the same specimens were printed using identical printing parameters. The nozzle temperature was set at 215 °C, and the bed temperature was set at 60 °C, which are common printing temperatures for PLA filament. Furthermore, to ensure satisfying extrudability, a 0.8 mm nozzle and 0.3 mm layer height were employed. Additionally, the specimens were printed with a 100% fill density and concentric fill pattern for infill. The printed specimens included: 1. Specimens for tensile testing following ASTM D638 Standard [50], Type V [50] 2. Specimens for Dynamic Mechanical Analysis (DMA) with dimensions of 50 × 5 × 1 mm, and 3. Specimens for antioxidant properties assessment with dimensions of 10 × 10 × 0.8 mm. Figure 4 displays the developed filaments, the 3D-printed specimens, and the equipment used.

### 2.5. Materials Characterization

#### 2.5.1. Determination of Tomato Stem Powder (TSP) Composition

The determination of TSP structural components (cellulose, hemicellulose, and lignin) ratio was carried out by biomass fractionation via hydrothermal and solvothermal (organosolv) treatment.

The hydrothermal treatment of TSP was conducted first to separate the water-soluble fraction of hemicellulose. The process was performed in a lab scale autoclave batch reactor (Parr, Model 4563) with a total volume of 600 mL according to previous methodology [51]. TSP particles were dispersed in deionized H_2_O at a Liquid to Solids Ratio, LSR = 15 and heated at 220 °C for 15 min under stirring (400 rpm) with heating rate ~7 °C/min. Next, the reactor was cooled to room temperature and the solid fractions of the reaction mixture were separated from the liquid phase via vacuum filtration, washed with deionized H_2_O, air dried overnight and 4 h at 105 °C, and finally weighed to calculate the mass of the remaining cellulose and lignin mixture.

Organosolv lignin and cellulose were isolated next via solvothermal treatment [52] of previously hydrothermally treated TSP particles using ethanol/water mixture. More specifically, 20 g of TSP were mixed with a solution of ethanol and deionized H_2_O (60:40 *v*/*v*) in a 600 mL stirred autoclave batch reactor and heated at 190 °C for 60 min, with heating rate ~7 °C/min and stirring speed 200 rpm. Afterwards, the reactor was quenched, and the resulting slurry was vacuum filtrated. The recovered solids (cellulose fraction) were washed with 200 mL deionized H_2_O. Additional 200 mL of deionized water were used for the precipitation of lignin from the filtrate. The precipitated, organosolv lignin was recovered with centrifugation and dried at 80 °C under vacuum.

#### 2.5.2. Microscopy

A Jenoptik (Jena, Germany) ProgRes GRYPHAX Altair camera attached to a ZEISS (Oberkochen, Germany) SteREO Discovery V20 microscope, and Gryphax image capturing software (GRYPHAX Version 2.2.0.1234 WINDOWS 64 Bit) was used for microscopy observations. Scanning Electron Microscopy (SEM) images were captured using a JEOL (Tokyo, Japan) 2011 (JMS-840) electron microscope, equipped with an Oxford (Abingdon, UK) ISIS 300 energy-dispersive X-ray (EDX) micro-analytical system. Every specimen was positioned on the holder and coated with carbon to enhance the conductivity of the electron beam. The images were taken under an accelerating voltage of 5 kV, a probe current of 45 nA, and a counting time of 60 s.

#### 2.5.3. Fourier-Transform Infrared Spectroscopy (FTIR)

FTIR spectra of the specimens were recorded utilizing an IRTracer-100 (Shimadzu, Japan) equipped with a QATR™ 10 Single-Reflection ATR Accessory with a Diamond Crystal. The spectra were collected in the range from 450 to 4000 cm^−1^ at a resolution of 2 cm^−1^ (total of 16 co-added scans), while the baseline was corrected and converted into absorbance mode.

#### 2.5.4. N_2_ Physisorption

The N_2_ porosimetry measurement of TSP specimen was conducted by obtaining the adsorption/ desorption isotherms at −196 °C on an Automatic Volumetric Sorption Analyzer (Autosorb-1MP, Quantachrome Instruments, Boynton Beach, FL, USA). The specimen was outgassed at 90 °C and 1.33 × 10^−4^ Pa for a minimum of 18 h prior to analysis. The specific surface area (SBET) was estimated by the multipoint BET method using the adsorption isotherm points in the range of 0.05 < P/Po < 0.20 and the total pore volume (V_p_) was determined by the adsorbed nitrogen at P/Po = 0.99.

#### 2.5.5. Thermal Gravimetric Analysis (TGA)

The thermal stability of TSP was determined via TGA, which was conducted using a Netzsch STA 449F5 instrument (Netzsch Group, Selb, Germany). TSP was placed in alu-mina crucible and was heated under a 50 mL/min flow of N_2_ and a heating rate of 10 K/min in the temperature range of 25–950 °C (composites).

#### 2.5.6. Mechanical Testing

Tensile testing evaluations were conducted utilizing a Shimadzu EZ Test Tensile Tester, Model EZ-LX, equipped with a 2 kN load cell, following the ASTM D638 standard [50] at a crosshead speed of 5 mm/min. For the testing, 3D-printed dumbbell-shaped tensile type V test specimens were employed. Each specimen underwent at least five separate assessments, with the resulting data averaged to derive the mean values for Young’s modulus, stress at break, and elongation at break. Flexural properties were measured using the same instrument, following the ASTM D790 standard [53] at a crosshead speed of 5 mm/min, using at least five specimens for each specimen.

#### 2.5.7. Dynamic Mechanical Analysis (DMA)

Dynamic mechanical analysis was used to measure the thermomechanical properties of the 3D-printed composites using a Perkin Elmer Diamond DMA analyzer. The bending method was applied with an oscillation frequency of 1 Hz and heating rate of 3 °C/min. The specimens were tested from 25 to 80 °C with 10 mN applied force. The specimens had a rectangular shape with dimensions of 20 × 13 × 3 mm and one measurement was conducted for each specimen.

#### 2.5.8. Antioxidant Properties Assessment

The antioxidant activity of the composites was assessed by observing the reduction rate of the DPPH radical in the presence of printed specimens using UV-Vis spectroscopy. This method determines the ability of a substance to scavenge free radicals by monitoring the decrease in absorbance of a DPPH solution after it is incubated with the test specimen. A 0.079 mM DPPH solution in EtOH was prepared and kept in the dark at room temperature for 16 h. Films with identical dimensions (1 cm × 1 cm) were immersed in 3 mL of the DPPH/EtOH solution at room temperature and stored in the dark. The antioxidant capacity of the composites was determined by measuring the absorption decay at 517 nm at regular time intervals using UV-Vis spectroscopy. The remaining DPPH content in the solution was calculated using Equation (1):

Residual DPPH content (%) = 100 − 100(A_0_ − A_1_/A_0_) (1)

where A_0_ represents the absorbance of the control specimen, and A_1_ is the absorbance in the presence of the materials.

## 3. Results and Discussion

### 3.1. Tomato Stem Powder (TSP) Composition

Biomass consists mainly of cellulose, hemicellulose, and lignin (structural components), as well as ash and extractives (nonstructural components). According to the calculations performed after the hydrothermal and solvothermal treatment in the present work, the TSP composition is cellulose 38%, hemicellulose 58%, lignin 6%, and nonstructural components 1%. This is a typical composition for some plant species, especially for the parts that are discarded as waste [54,55].

### 3.2. Characterization of Tomato Stem Powder (TSP)

The waste of tomato agriculture accounts for 10–15 wt.% of the total production volume, and while skin, seeds, and tomato pomace are commonly used as compost or animal feed, the lignocellulosic waste, i.e., the stems, are either discarded or incinerated. Stem waste results from both pruning and final cropping. While the fruit’s byproducts are well known for their bioactive compounds and potential exploitation in the pharmaceutical industry, the nonedible waste remains underutilized.

**Figure 5:**
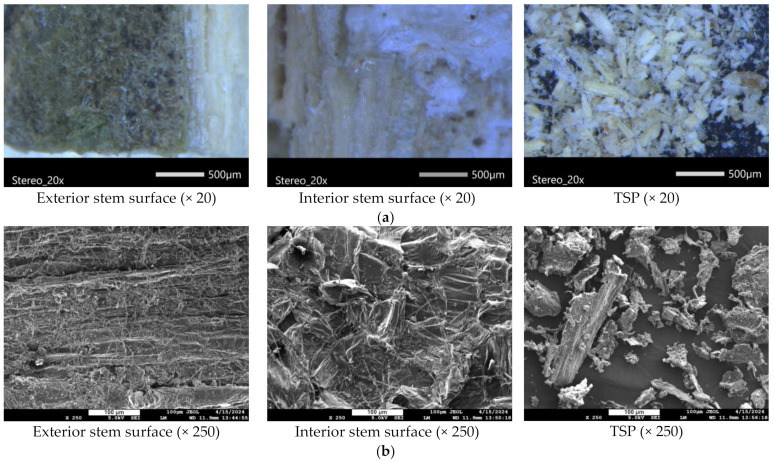

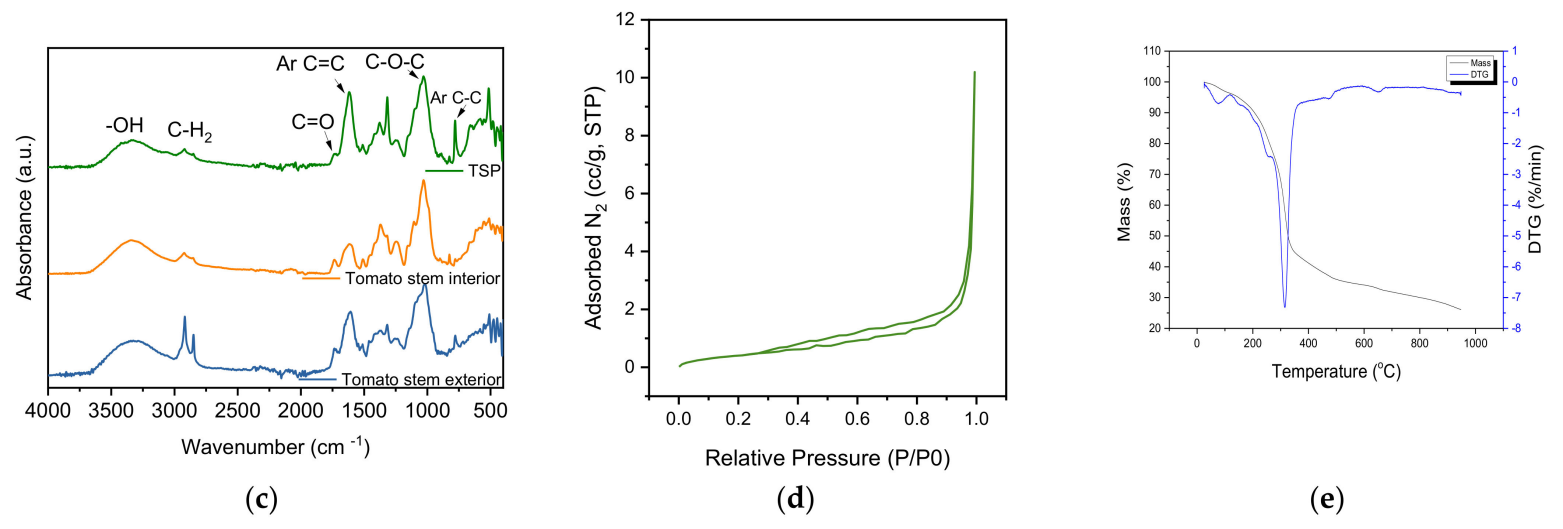
Characterization of tomato stem and TSP: (**a**) Optical microscopy images, (**b**) SEM images and (**c**) FTIR-ATR spectra, (**d**) N_2_ adsorption/desorption isotherm, and (**e**) TGA of tomato stem and its powder.

The as-received stems (Figure 2) were cut into small pieces followed by knife milling, which resulted in a fine powder. Optical microscopy images of the tomato stems before and after milling is shown in Figure 5a. As can be seen, the stems had a green-coloured epidermis, and a softer and lighter centre of the stem known as the pith. The TSP particles vary in shape, size, and texture. The white parts of TSP consist of cellulose and hemicellulose, while the green/brown particles consist of lignin and probably some pigment, e.g., chlorophyll. The microstructure of the tomato stems is shown in the SEM images of Figure 5b. The epidermis (exterior surface) is covered in trichomes, and the pith (interior surface) in cellulose fibres. The powder fraction (TSP) that was used to produce printed PLA composites was separated using a 150 μm sieve and, as a result, their particle size ranged from slightly larger than 150 μm to much smaller particles. The particles vary in shape as well, having both elongated irregular morphologies as well as small, powder-like pieces. TSP does not have the surface roughness of the stem before milling and no visible fibre-like structures. This indicates that the milling process homogenized the microstructure of the stems and decreased the visible surface porosity. Previous studies have shown that the biochemical composition of tomato biomass is affected by the sieve size used after milling. More specifically, when the sieve size is smaller than 250 μm, the glucose and xylose content increase, indicating an increase in cellulose content as well, which often correlates with mechanical performance [56].

The chemical structure of the tomato stem and the TSP was examined with FTIR-ATR spectroscopy, and the resulting spectra are presented in Figure 5c. Tomato stems contain mainly holocellulose, α-cellulose and lignin [57,58] as well as pectin and waxes (i.e., cutin-embedded polysaccharides) on the epidermis that protect the plant from water loss and provide a barrier against pathogens and environmental stresses. The O-H stretching vibrations of the polysaccharide’s hydroxyls appear at 3000–3600 cm^−1^. At 2918 cm^−1^ and 2850 cm^−1^, the C-H asymmetric and symmetric stretching of methylene groups is present. These two peaks are sharp in the spectrum of the stem’s exterior (blue line), due to the presence of cutin, which are made of long-chain (mainly C16 and C18) hydroxy fatty acids linked together by ester bonds. The stretch vibrations of these ester bonds, as well as the esters of hemicellulose and pectin absorb at 1735 cm^−1^. The skeletal stretching vibrations of aromatic rings, which exist in lignin and other phenolic compounds of the tomato plant, appear at 1610–1620 cm^−1^, while the aromatic C-H rocking vibrations appear at 780 cm^−1^. The peak of the C-O-C vibrations of the glycosidic bond of the polysaccharides is reflected upon a strong peak at 1030 cm^−1^. As expected, the TSP spectrum contains all the peaks of both the interior and the exterior of the stem, but with stronger absorbance in the fingerprint region, which can be associated with the increased surface area of the smaller particles, thus exposing more of the characteristic groups and resulting in a stronger FTIR signal.

The N_2_ physisorption experiments resulted in the adsorption/ desorption isotherms of Figure 5d. As expected, the knife-milled TSP is a nonporous material and the interparticle voids have 0.016 cc/g pore volume (V_p_). The calculated specific surface area (S_BET_) corresponds to the external surface area of the particles and equals to 1.7 m^2^/g.

The TGA of TSP revealed that the biomass-derived substance is thermally stable up to 279.6 °C, which is higher than the highest temperature during filament development (175 °C), as well as the highest temperature during 3D printing (215 °C nozzle temperature).

### 3.3. Composite’s Morphology

**Figure 6:**
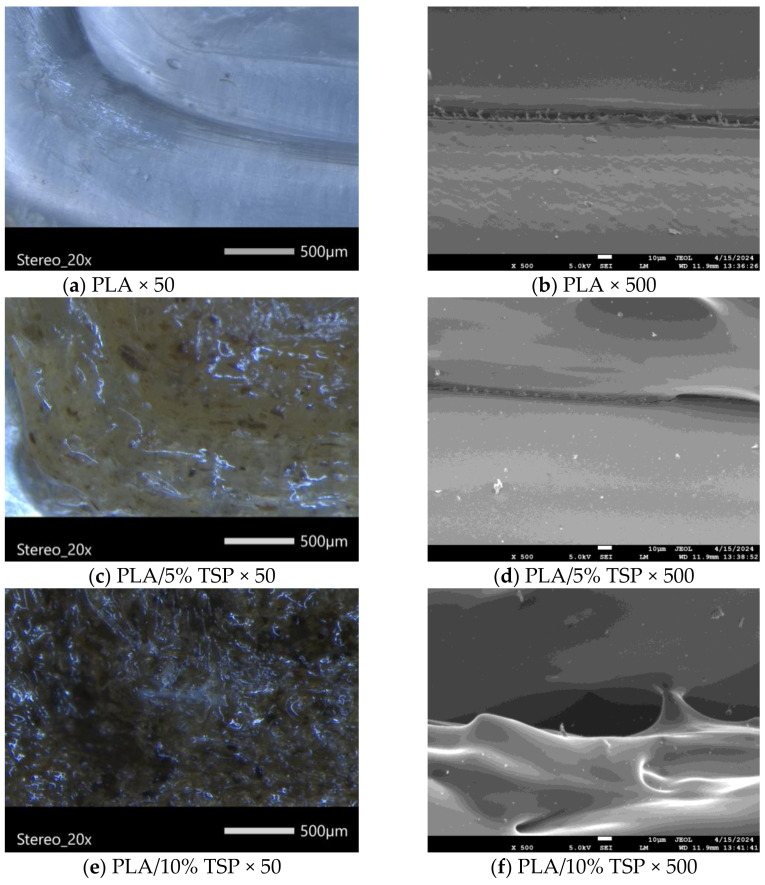
Stereoscope and SEM images of 3D-printed PLA and its composites with TSP.

The morphology of the 3D-printed structure was examined using optical microscopy and SEM (Figure 6). In the stereoscope images (left column of Figure 6) the colouration of the composites due to the presence of TPS is obvious. Additionally, some darker spots that indicate the presence of aggregates appear and the texture is rougher in comparison with neat PLA, but no printing defects are visible. In the SEM images (right column of Figure 6), there are no visible pores on the surface of the composites, which suggests the lack of phase separation, likely a result of the presence of the chain extender Joncryl. During melt compounding in the filament maker, the epoxy groups of Joncryl undergo ring opening and form covalent bonds with the -OH groups of the TSP as well as the terminal -OH groups of PLA, which could have contributed to the lack of visible phase separation [23]. The bonding is also visible in the SEM images. The filament of neat PLA (Figure 6b) shows good bonding after printing with a small visible gap, and small fibre-like structures protruding. Both the gap and the fibre-like structures could be caused by the typical shrinking and deformations of PLA observed after its thermal treatment in the 3D printer [59]. The composites (Figure 6d,f) have larger voids in the space between the filament. During printing, the PLA filament softens and the layers bond. When the TSP additive is added, the heat transfer in the PLA matrix becomes more difficult since they are not heat conductors. The increase in the surface roughness is also visible in the SEM image of Figure 6f, in agreement with the macroscopic observations.

### 3.4. Tensile Strength Measurements

**Figure 7:**
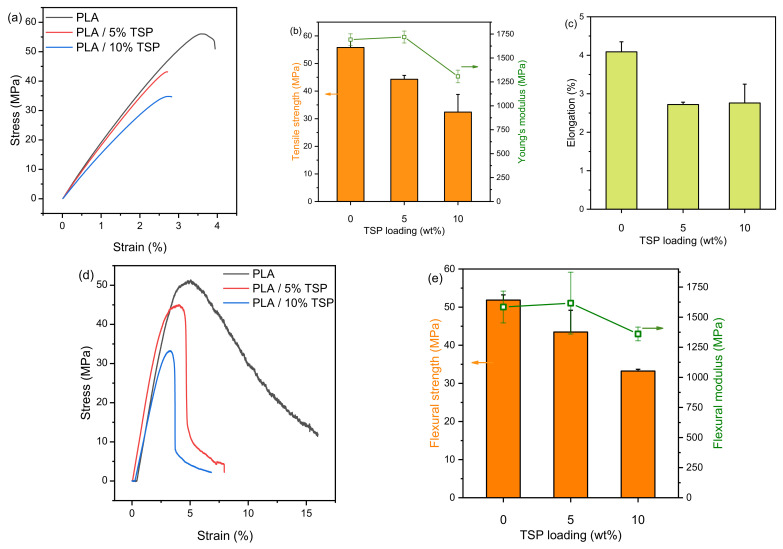
Mechanical properties of 3D-printed materials. (**a**–**c**) tensile strength, and (**d**,**e**) flexural strength.

Figure 7, shows the tensile and flexural test results of the 3D-printed materials. The stress–strain test results are presented in Table 1. The indicative tensile stress–strain curves of pristine and composite PLA specimens (Figure 7a) exhibited an initial linear elastic behaviour, followed by a nonlinear region until the maximum stress was reached and failure occurred. This behaviour is typical of hard and brittle materials. The average maximum stress and Young’s modulus of the pristine and composite PLA specimens are presented in Figure 7b, while the average strain to failure is in Figure 7c. It is noted that the mechanical properties of PLA are decreased after the addition of TSP, while the specimen with 5% TSP has slightly better properties compared to the specimen with 10% TSP. This decrease is related to the void observed in the SEM image of Figure 6f, which can cause lack of effective stress transfer and premature failure. Although it has been reported that the 3D printing and extrusion processes could cause mechanical properties reduction due to increased porosity [60] molecular weight reduction [61,62] and particle agglomeration [63], it appears that the drop in mechanical properties, especially the deformation as described by the Young’s modulus, is kept to a minimum, especially for the specimen with 5% TPS loading. In this work, the reduction of molecular weight was avoided using the chain extender [49], which could have contributed to retaining the tensile properties at 5 wt.% TSP content.

The effect of the additive is more pronounced in the case of flexural strength; as shown in Figure 7d,e, neat PLA has a flexural strength of 51.85 ± 1.37 MPa [64], which decreases with increasing TSP content. Thus, the composites are less able to resist deformation under bending and in contrast with neat PLA, the specimens broke during testing. This is related to both the rigid nature of the additive as well as the presence of voids between the printed layers that could be points of premature failure. The flexural modulus is not significantly affected at 5 wt.% of TSP, and decreases at 10 wt.%, in agreement with Young’s modulus trend, which means that adding 10 wt.% of TSP in PLA reduces its stiffness. Decreased stiffness was also obtained after the addition of 5–15 wt.% of buckwheat husks particles in 3D-printed PLA [23].

### 3.5. Dynamic Mechanical Analysis (DMA) Measurements

**Figure 8:**
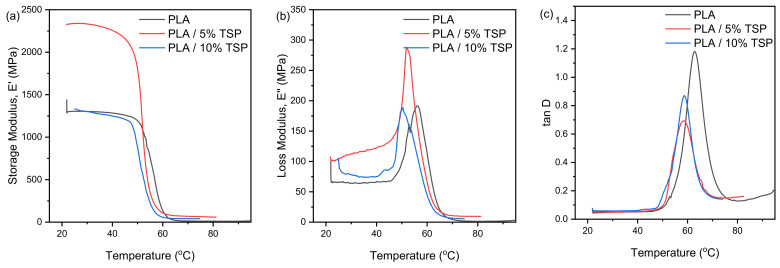
DMA of 3D-printed materials, (**a**) storage modulus, (**b**) loss modulus, and (**c**) damping factor tanD.

The glass–rubber transition of printed materials was studied via DMA. The storage modulus, E′, and damping factor, tanD, curves as a function of temperature are shown in Figure 8. Storage modulus of pristine and composite PLA specimens (Figure 8a) has high values at low temperature (glassy region), followed by a sharp drop (glass transition), and a low-value plateau at higher temperatures (rubbery region). Comparing the viscoelastic profile of the materials, their behaviour, as resistance to deformation, is different to tensile tests. More specifically, at 25 °C, the PLA/5% TSP has the highest storage modulus of all the specimens, while the pristine polymer and the composite with 10% of TSP have similar storage modulus values. On the contrary, at 75 °C, the 5% TSP composite has the highest storage modulus, followed by the 10% composite, while the pristine polymer has the lowest storage modulus. The peak intensity of loss modulus represents the melt viscosity of a polymer (Figure 8b). As can be seen, the loss modulus curves follow the same trend to the storage modulus curves. The higher values are reported for the 5% TSP composite, while the loss modulus curves of the neat polymer and 10% TSP composite are of the same magnitude. The higher E′ and E″ values of the 5% TSP composite may be attributed to better dispersion of the additive in the PLA matrix [65]. The damping factor tanD (Figure 8c) is defined as the ratio of the loss modulus, E″, to the storage modulus, E′, and the shape of the curve is correlated with the glass transition of the material. In this work, the glass transition temperature, Tg, of the materials is determined by the peak of each curve. Therefore, it is observed that the addition of TSP results in a slight (less than 5 °C) decrease of the Tg but varying the TSP concentration between 5 and 10% seems to bear no ef-fect (PLA 62.9 °C, 5% TSP 57.6 °C, and 10% TSP 58.3 °C). Table 2 presents the viscoelastic properties of the pristine and composite PLA specimens.

### 3.6. Antioxidant Properties Assessment

**Figure 9:**
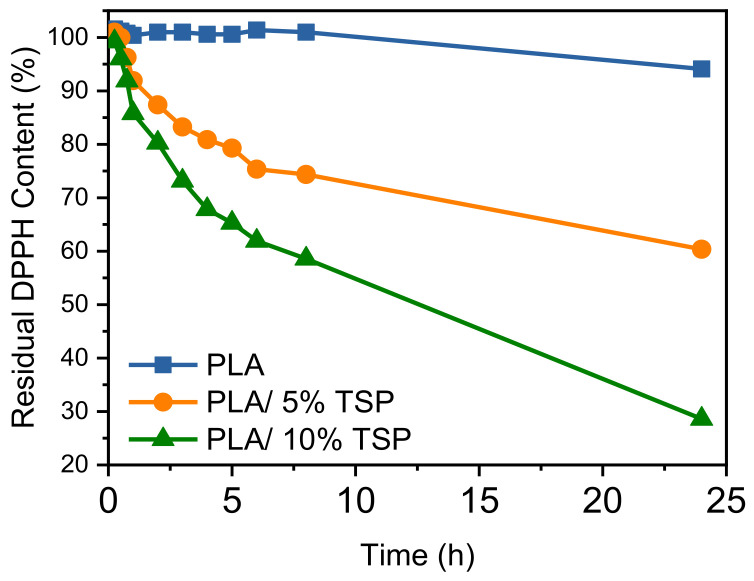
Residual DPPH content after immersion of PLA and its composites with TSP.

The kinetics of DPPH reduction are presented in Figure 9. Residual DPPH content after immersion of PLA and its composites with TSP. Neat PLA does not reduce the DPPH content after immersion for up to 24 h. On the other hand, the composites exhibit antioxidant activity, witnessed by the decrease in DPPH. The antioxidant activity is increases as the TSP content increases. By adding only 10 wt.% of TSP, the residual DPPH content was as low as ~30%. Tomato crop remains contain bioactive compounds, including quercetin-3-O-rutinoside, which imparts them with antioxidant activity [36]. Thus, the phenolic compounds of the stems used herein retained their structure and stability after the drying, milling, compounding and 3D printing procedures, making the pretreatment of tomato waste to isolate bioactive compounds unnecessarily. The tomato stems are rarely valorised in the literature, in contrast to other parts of the plant, despite their good potential. Manríquez-Altamirano et al. concluded that tomato stems could find applications as higher quality ecomaterial without losing the benefits of its processing and use locally in fences and trellises, packaging materials, biodegradable pots, and boards/panels [66]. The herein identified antioxidant properties of the PLA/TSP composites open up new options in high-added-value applications of tomato stems.

## 4. Conclusions

The present study showcases circular economy promotion in agricultural waste management through additive manufacturing technologies. This innovative approach explores the integration of tomato stem powder into polymeric matrix materials, particularly PLA. Three FFF PLA-based filaments were fabricated: two with tomato stem powder (5% and 10% wt.%) and one with PLA alone as a reference. All filaments included Joncryl as a chain extender for enhanced printability. The findings demonstrate satisfactory printability by integrating tomato stem powder into the polymer matrix.

The study examined the morphology, mechanical properties, and functional characteristics of 3D-printed composite materials. Morphological analysis revealed a lack of visible phase separation. Mechanical testing showed consistent tensile properties with a 5% TSP addition, while flexural strength decreased, possibly due to void formation between the printed filaments. The composite with a 5% TSP had higher mechanical and thermomechanical properties compared to the composite with a 10% TSP. DMA analysis highlighted changes in storage modulus and damping factor with TSP addition. Importantly, the composites exhibited antioxidant activity, enhancing with increased TSP content.

The obtained results highlight the potential of utilizing agricultural waste in FFF technology, paving the way for the creation of various closed-loop systems in the agricultural sector and contributing to greener agricultural waste management. Current challenges in valorizing agricultural and food waste in FFF are the deterioration of the mechanical performance and its compatibility with common plastics. Herein, we demonstrated that reducing the particle size of the waste and exploiting standard chain extenders could be a means to mitigate these problems for low additive contents. Future studies can investigate enhancing mechanical performance by optimizing various parameters. These include the blending process of agricultural waste and the polymer matrix, controlling particle size distribution, and incorporating chemicals like stabilizers and compatibilizers to better integrate agricultural waste additives into the polymer matrix materials. Concerning the applicability of the PLA/TSP composites, the printed material has suitable properties (physicochemical, form, texture) for numerous applications (e.g., structural components, interior design) across various industry sectors (e.g., furniture, wood industries). Moreover, such composites can be used by mass-production 3D printers without necessitating complex intermediate processing stages of raw materials, thereby reducing the amount of plastic used.

## Figures and Tables

**Table 1:** Mechanical properties of 3D-printed PLA/TSP composites.

Mechanical Properties					
TSP Loading (wt. %)	Tensile Strain at Break (%)	Tensile Stress at Maximum Load (mpa)	Young’s Modulus (MPa)	Flexural Strength (MPa)	Flexural Modulus (MPa)
0	4.09 ± 0.26	55.8 ± 3.4	1692 ± 61	51.85 ± 1.37	1584.03 ± 131.59
5	2.72 ± 0.06	44.3 ± 1.4	1719 ± 62	43.47 ± 5.70	1616.44 ± 255.93
10	2.76 ± 0.49	32.4 ± 6.4	1307 ± 65	33.23 ± 0.47	1360.41 ± 56.25

**Table 2:** Viscoelastic properties of 3D-printed PLA/TSP composites.

Viscoelastic Properties			
TSP Loading (wt. %)	Storage Modulus, E′, at 25 °C (MPa)	Storage Modulus, E′, at 75 °C (MPa)	Glass Transition Temperature, Tg (°C)
0	1305	12	62.9
5	2338	65	57.6
10	1332	40	58.3

## Data Availability

All the data are included in the manuscript.

## References

[1] Aloini D., Dulmin R., Mininno V., Stefanini A., Zerbino P. (2020). Driving the Transition to a Circular Economic Model: A Systematic Review on Drivers and Critical Success Factors in Circular Economy. Sustainability.

[2] Padilla-Rivera A., Russo-Garrido S., Merveille N. (2020). Addressing the Social Aspects of a Circular Economy: A Systematic Literature Review. Sustainability.

[3] Romani A., Rognoli V., Levi M. (2021). Design, Materials, and Extrusion-Based Additive Manufacturing in Circular Economy Contexts: From Waste to New Products. Sustainability.

[4] Kara S., Hauschild M., Sutherland J., McAloone T. (2022). Closed-Loop Systems to Circular Economy: A Pathway to Environmental Sustainability?. CIRP Ann..

[5] Campbell-Johnston K., Vermeulen W.J.V., Reike D., Brullot S. (2020). The Circular Economy and Cascading: Towards a Framework. Resour. Conserv. Recycl. X.

[6] Reike D., Vermeulen W.J.V., Witjes S. (2018). The Circular Economy: New or Refurbished as CE 3.0?—Exploring Controversies in the Conceptualization of the Circular Economy through a Focus on History and Resource Value Retention Options. Resour. Conserv. Recycl..

[7] Razmjooei D., Alimohammadlou M., Ranaei Kordshouli H.-A., Askarifar K. (2024). A Bibliometric Analysis of the Literature on Circular Economy and Sustainability in Maritime Studies. Environ. Dev. Sustain..

[8] Khan F.A., Tomar A., Agarwal Y.K., Shukla H.O., Baskar C., Ramakrishna S., Baskar S., Sharma R., Chinnappan A., Sehrawat R. (2022). Agricultural Solid Waste Management: An Approach to Protect the Environment and Increase Agricultural Productivity. Handbook of Solid Waste Management: Sustainability through Circular Economy.

[9] Mettu S., Halder P., Patel S., Kundu S., Shah K., Yao S., Hathi Z., Ong K.L., Athukoralalage S., Choudhury N.R. (2020). Valorisation of Agricultural Waste Residues. Waste Valorisation.

[10] Iqbal N., Agrawal A., Dubey S., Kumar J., Iqbal N., Agrawal A., Dubey S., Kumar J. (2020). Role of Decomposers in Agricultural Waste Management. Biotechnological Applications of Biomass.

[11] El-Ramady H., El-Henawy A., Amer M., Omara A.E.-D., Elsakhawy T., Elbasiouny H., Elbehiry F., Abou Elyazid D., El-Mahrouk M. (2020). Agricultural Waste and Its Nano-Management: Mini Review. Egypt. J. Soil Sci..

[12] Ayilara M.S., Olanrewaju O.S., Babalola O.O., Odeyemi O. (2020). Waste Management through Composting: Challenges and Potentials. Sustainability.

[13] Waqas M., Hashim S., Humphries U.W., Ahmad S., Noor R., Shoaib M., Naseem A., Hlaing P.T., Lin H.A. (2023). Composting Processes for Agricultural Waste Management: A Comprehensive Review. Processes.

[14] Jain N. (2014). Emission of Air Pollutants from Crop Residue Burning in India. Aerosol Air Qual. Res..

[15] Haque F., Fan C., Lee Y.-Y. (2023). From Waste to Value: Addressing the Relevance of Waste Recovery to Agricultural Sector in Line with Circular Economy. J. Clean. Prod..

[16] Pinho A.C., Amaro A.M., Piedade A.P. (2020). 3D Printing Goes Greener: Study of the Properties of Post-Consumer Recycled Polymers for the Manufacturing of Engineering Components. Waste Manag..

[17] John M.J., Dyanti N., Mokhena T., Agbakoba V., Sithole B. (2021). Design and Development of Cellulosic Bionanocomposites from Forestry Waste Residues for 3D Printing Applications. Materials.

[18] Scaffaro R., Maio A., Gulino E.F., Alaimo G., Morreale M. (2021). Green Composites Based on PLA and Agricultural or Marine Waste Prepared by FDM. Polymers.

[19] Lanzotti A., Martorelli M., Maietta S., Gerbino S., Penta F., Gloria A. (2019). A Comparison between Mechanical Properties of Specimens 3D Printed with Virgin and Recycled PLA. Procedia CIRP.

[20] Pechlivani E.M., Papadimitriou A., Pemas S., Ntinas G., Tzovaras D. (2023). IoT-Based Agro-Toolbox for Soil Analysis and Environmental Monitoring. Micromachines.

[21] Scaffaro R., Citarrella M.C., Catania A., Settanni L. (2022). Green Composites Based on Biodegradable Polymers and Anchovy (*Engraulis Encrasicolus*) Waste Suitable for 3D Printing Applications. Compos. Sci. Technol..

[22] Dey T., Bhattacharjee T., Nag P., Ritika, Ghati A., Kuila A. (2021). Valorization of Agro-Waste into Value Added Products for Sustainable Development. Bioresour. Technol. Rep..

[23] Andrzejewski J., Grad K., Wiśniewski W., Szulc J. (2021). The Use of Agricultural Waste in the Modification of Poly(Lactic Acid)-Based Composites Intended for 3D Printing Applications. The Use of Toughened Blend Systems to Improve Mechanical Properties. J. Compos. Sci..

[24] Bahcegul E.G., Bahcegul E., Ozkan N. (2020). 3D Printing of Hemicellulosic Biopolymers Extracted from Lignocellulosic Agricultural Wastes. ACS Appl. Polym. Mater..

[25] Calì M., Pascoletti G., Gaeta M., Milazzo G., Ambu R. (2020). New Filaments with Natural Fillers for FDM 3D Printing and Their Applications in Biomedical Field. Procedia Manuf..

[26] Fico D., Rizzo D., De Carolis V., Esposito Corcione C. (2024). Bio-Composite Filaments Based on Poly(Lactic Acid) and Cocoa Bean Shell Waste for Fused Filament Fabrication (FFF): Production, Characterization and 3D Printing. Materials.

[27] Pemas S., Xanthopoulou E., Terzopoulou Z., Konstantopoulos G., Bikiaris D.N., Kottaridi C., Tzovaras D., Pechlivani E.M. (2023). Exploration of Methodologies for Developing Antimicrobial Fused Filament Fabrication Parts. Materials.

[28] Ahmad M.N., Ishak M.R., Mohammad Taha M., Mustapha F., Leman Z. (2023). A Review of Natural Fiber-Based Filaments for 3D Printing: Filament Fabrication and Characterization. Materials.

[29] Woern A.L., Byard D.J., Oakley R.B., Fiedler M.J., Snabes S.L., Pearce J.M. (2018). Fused Particle Fabrication 3-D Printing: Recycled Materials’ Optimization and Mechanical Properties. Materials.

[30] Reich M.J., Woern A.L., Tanikella N.G., Pearce J.M. (2019). Mechanical Properties and Applications of Recycled Polycarbonate Particle Material Extrusion-Based Additive Manufacturing. Materials.

[31] Acevedo M., Royano L., Parralejo A.I., Cabanillas J., González J.F., González J., da Costa Sanches Galvão J.R., Duque de Brito P.S., dos Santos Neves F., da Silva Craveiro F.G., de Amorim Almeida H., Correia Vasco J.O., Pires Neves L.M., de Jesus Gomes R., de Jesus Martins Mourato S., Santos Ribeiro V.S. (2021). 3D Printing Filaments from Kenaf, Poplar and Agricultural Residues. Proceedings of the 1st International Conference on Water Energy Food and Sustainability (ICoWEFS 2021), Leiria, Portugal, 10–12 May 2021.

[32] Harshwardhan K., Upadhyay K. (2017). Effective Utilization of Agricultural Waste: Review. J. Fundam. Renew. Energy Appl..

[33] Puglia D., Pezzolla D., Gigliotti G., Torre L., Bartucca M.L., Del Buono D. (2021). The Opportunity of Valorizing Agricultural Waste, Through Its Conversion into Biostimulants, Biofertilizers, and Biopolymers. Sustainability.

[34] Terzopoulou Z.N., Papageorgiou G.Z., Papadopoulou E., Athanassiadou E., Alexopoulou E., Bikiaris D.N. (2015). Green Composites Prepared from Aliphatic Polyesters and Bast Fibers. Ind. Crops Prod..

[35] Pardalis N., Xanthopoulou E., Zamboulis A., Bikiaris D. (2024). Olive Stone as a Filler for Recycled High-Density Polyethylene: A Promising Valorization of Solid Wastes from Olive Oil Industry. Sustain. Chem. Environ..

[36] Añibarro-Ortega M., Pinela J., Ćirić A., Martins V., Rocha F., Soković M.D., Barata A.M., Carvalho A.M., Barros L., Ferreira I.C.F.R. (2020). Valorisation of Table Tomato Crop By-Products: Phenolic Profiles and In Vitro Antioxidant and Antimicrobial Activities. Food Bioprod. Process..

[37] Food and Agriculture Organization of the United Nations Crop, Livestock and Food. http://www.fao.org/food-agriculture-statistics/data-release/crop-livestock-and-food/en/.

[38] Jeguirim S.E., Jeguirim M., Zorpas A. (2022). CHAPTER FIVE—Tomato Wastes Valorization for Bio-Based Materials Production. Tomato Processing by-Products.

[39] Fritsch C., Staebler A., Happel A., Cubero Márquez M.A., Aguiló-Aguayo I., Abadias M., Gallur M., Cigognini I.M., Montanari A., López M.J. (2017). Processing, Valorization and Application of Bio-Waste Derived Compounds from Potato, Tomato, Olive and Cereals: A Review. Sustainability.

[40] Kraiem N., Lajili M., Limousy L., Said R., Jeguirim M. (2016). Energy Recovery from Tunisian Agri-Food Wastes: Evaluation of Combustion Performance and Emissions Characteristics of Green Pellets Prepared from Tomato Residues and Grape Marc. Energy.

[41] Boccia F., Di Donato P., Covino D., Poli A. (2019). Food Waste and Bio-Economy: A Scenario for the Italian Tomato Market. J. Clean. Prod..

[42] Marinos S., Terpsithea P., Hamdi H., Michail T., Zorpas A.A., Agapios A., Jeguirim M., Zorpas A. (2022). CHAPTER SIX—Biochar Production from the Pyrolysis of Tomato Processing Residues. Tomato Processing by-Products.

[43] Ji A., Zhang S., Bhagia S., Yoo C.G., Ragauskas A.J. (2020). 3D Printing of Biomass-Derived Composites: Application and Characterization Approaches. RSC Adv..

[44] Hassan M., Mohanty A.K., Misra M. (2024). 3D Printing in Upcycling Plastic and Biomass Waste to Sustainable Polymer Blends and Composites: A Review. Mater. Des..

[45] Shi S., Jiang Y., Ren H., Deng S., Sun J., Cheng F., Jing J., Chen Y. (2024). 3D-Printed Carbon-Based Conformal Electromagnetic Interference Shielding Module for Integrated Electronics. Nano-Micro Lett..

[46] Ruan J., Chang Z., Rong H., Alomar T.S., Zhu D., AlMasoud N., Liao Y., Zhao R., Zhao X., Li Y. (2023). High-Conductivity Nickel Shells Encapsulated Wood-Derived Porous Carbon for Improved Electromagnetic Interference Shielding. Carbon.

[47] Pechlivani E.M., Papadimitriou A., Pemas S., Giakoumoglou N., Tzovaras D. (2023). Low-Cost Hyperspectral Imaging Device for Portable Remote Sensing. Instruments.

[48] Bhagia S., Bornani K., Agrawal R., Satlewal A., Ďurkovič J., Lagaňa R., Bhagia M., Yoo C.G., Zhao X., Kunc V. (2021). Critical Review of FDM 3D Printing of PLA Biocomposites Filled with Biomass Resources, Characterization, Biodegradability, Upcycling and Opportunities for Biorefineries. Appl. Mater. Today.

[49] Grigora M.-E., Terzopoulou Z., Tsongas K., Klonos P., Kalafatakis N., Bikiaris D.N., Kyritsis A., Tzetzis D. (2021). Influence of Reactive Chain Extension on the Properties of 3D Printed Poly(Lactic Acid) Constructs. Polymers.

[50] (2014). Standard Test Method for Tensile Properties of Plastics.

[51] Margellou A.G., Pappa C.P., Psochia E.A., Petala M.D., Triantafyllidis K.S. (2023). Mild Isolation and Characterization of Surface Lignin from Hydrothermally Pretreated Lignocellulosic Forestry and Agro-Industrial Waste Biomass. Sustain. Chem. Pharm..

[52] Margellou A.G., Lazaridis P.A., Charisteidis I.D., Nitsos C.K., Pappa C.P., Fotopoulos A.P., Van den Bosch S., Sels B.F., Triantafyllidis K.S. (2021). Catalytic Fast Pyrolysis of Beech Wood Lignin Isolated by Different Biomass (Pre)Treatment Processes: Organosolv, Hydrothermal and Enzymatic Hydrolysis. Appl. Catal. A Gen..

[53] (2016). Standard Test Methods for Flexural Properties of Unreinforced and Reinforced Plastics and Electrical Insulating Materials.

[54] Riseh R.S., Vazvani M.G., Hassanisaadi M., Thakur V.K. (2024). Agricultural Wastes: A Practical and Potential Source for the Isolation and Preparation of Cellulose and Application in Agriculture and Different Industries. Ind. Crops Prod..

[55] Singh R.P., Sarkar A. (2015). Waste Management: Challenges, Threats and Opportunities.

[56] Bourmaud A., Konschak K., Buffet C., Calatraba M., Rudolph A.L., Kervoëlen A., Gautherot B., Bonnin E., Beaugrand J. (2023). A Circular Approach for the Valorization of Tomato By-Product in Biodegradable Injected Materials for Horticulture Sector. Polymers.

[57] Uner B., Kömbeci K., Akgul M. (2016). The Utilization of Tomato Stalk in Fiber Production: NaOH and CaO Pulping Process. Wood Res..

[58] Güntekin E., Uner B., Karakus B. (2009). Chemical Composition of Tomato (*Solanum lycopersicum*) Stalk and Suitability in the Particleboard Production. J. Environ. Biol./Acad. Environ. Biol. India.

[59] Olam M. (2022). Determining of Process Parameters of the PLA/Titanium Dioxide/Hydroxyapatite Filament. Adv. Mater. Process. Technol..

[60] Liao Y., Liu C., Coppola B., Barra G., Di Maio L., Incarnato L., Lafdi K. (2019). Effect of Porosity and Crystallinity on 3D Printed PLA Properties. Polymers.

[61] Zhao P., Rao C., Gu F., Sharmin N., Fu J. (2018). Close-Looped Recycling of Polylactic Acid Used in 3D Printing: An Experimental Investigation and Life Cycle Assessment. J. Clean. Prod..

[62] Carrasco F., Pagès P., Gámez-Pérez J., Santana O.O., Maspoch M.L. (2010). Processing of Poly(Lactic Acid): Characterization of Chemical Structure, Thermal Stability and Mechanical Properties. Polym. Degrad. Stab..

[63] Garzon-Hernandez S., Garcia-Gonzalez D., Jérusalem A., Arias A. (2020). Design of FDM 3D Printed Polymers: An Experimental-Modelling Methodology for the Prediction of Mechanical Properties. Mater. Des..

[64] Li N., Li Y., Liu S. (2016). Rapid Prototyping of Continuous Carbon Fiber Reinforced Polylactic Acid Composites by 3D Printing. J. Mater. Process. Technol..

[65] Dong J., Li M., Zhou L., Lee S., Mei C., Xu X., Wu Q. (2017). The Influence of Grafted Cellulose Nanofibers and Postextrusion Annealing Treatment on Selected Properties of Poly(Lactic Acid) Filaments for 3D Printing. J. Polym. Sci. Part B Polym. Phys..

[66] Manríquez-Altamirano A., Sierra-Pérez J., Muñoz P., Gabarrell X. (2021). Identifying Potential Applications for Residual Biomass from Urban Agriculture through Eco-Ideation: Tomato Stems from Rooftop Greenhouses. J. Clean. Prod..

